# MiRNA-196-5p Promotes Proliferation and Migration in Cholangiocarcinoma via HAND1/Wnt/*β*-Catenin Signaling Pathway

**DOI:** 10.1155/2022/4599676

**Published:** 2022-04-12

**Authors:** Chao Liu, Yanli Li, Lichao Zhang, Pei Zhang, Ning Yu, Xuefang Liu, Huaihai Lu, Haiming Du, Senlin Hou

**Affiliations:** ^1^Hebei Medical University, China; ^2^The Second Hospital of Hebei Medical University, China

## Abstract

Accumulating evidence has indicated the crucial role of microRNA-196 in mediating tumor progression, while its significance in cholangiocarcinoma (CCA) remains unclear. In this study, we provided the first evidence that the expression level of miR-196-5p is elevated in both CCA cell lines and clinic specimen. MiR-196-5p inhibition notably suppressed cell proliferation as well as metastasis in CCA cell line HuCCT1. Furthermore, the interaction between miR-196-5p and its downstream molecule HAND1 was verified. Moreover, a series of rescue assay verified that both HAND1 and *β*-catenin silencing could reverse the abnormal elevated cell proliferation and migration brought by miR-196-5p elevation, indicating that HAND1/Wnt/*β*-catenin signaling pathway activation is essential for miR-196-5p to exert its roles. In summary, we successfully depict the oncogenic role of miR-196-5p in promoting cell proliferation and migration in CCA via HAND1/Wnt/*β*-catenin axis.

## 1. Introduction

As the second most common primary tumor in the liver, cholangiocarcinoma (CCA) is a highly aggressive tumor arising from bile duct epithelial cells with a relatively high incidence in Asia (more than 6 out of 10,000 habitants per year) [1, 2]. Despite that amounting efforts have been made in systemic and locoregional therapy for CCA, its current survival rate is still poor [3, 4]. One main factor that contributes to the poor prognosis is its atypical signs in early stage. Most CCA (approximately 70%) cases are initially diagnosed at an advanced stage with distant metastasis, making surgical resection (the only potential curative treatment) no longer possible [1, 5, 6]. Even worse, compared with many other malignant tumors, limited success has been made in finding chemotherapeutic targets for CCA. Therefore, further exploration of the molecular mechanisms underlying the pathogenesis of CCA is urgently needed to develop early diagnostic and potential therapeutic targets [7–9].

MiRNAs are short non-coding RNAs of 20-24 nucleotides that serve as critical posttranscriptional regulators of gene expression by binding to the 3′-untranslated region (3′-UTR) of their target mRNAs [10]. MiRNAs play a crucial role in regulating fundamental cellular processes [11]. In cancer, miRNAs exert their efforts by influencing numerous biological processes including proliferation, migration, apoptosis, cell cycle control, and differentiation [12, 13]. For instance, Huang et al. once reported that in CCA, multiple miRNAs including miR-21, miR-141, miR-200b, and let-7a family were involved in drug resistance [14]. Moreover, miR-204 has been proven to be a reliable tool for both CCA and gastric cancer diagnosis [15]. As for miR-196-5p, it has been reported to exert its oncogenic role in variant malignancies including breast cancer, colorectal cancer, oral cancer, and hepatocellular carcinoma [16–18]. However, to date, the involvement of miR-196-5p in cholangiocarcinoma pathogenesis has yet been fully elucidated.

HAND1 is known for its role in trophoblast giant cell differentiation and in cardiac morphogenesis by encoding heart and neural crest derivative expressed protein. Diseases associated with HAND1 mainly include congenital heart defects [19–21]. Recently, the role of HAND1 in tumorgenesis has been uncovered. In gastrointestinal stromal tumors, for instance, HAND1 has been reported to suppress cell growth [22]. Asuthkar et al. also demonstrated that Hand1 expression is observed to be downregulated in all subtypes of medulloblastoma and revealed the role of HAND1 in the context of epithelial-mesenchymal transition (EMT) [23]. Until now, not much is known about the identification, nor the role of HAND1 in CCA.

In our current work, we found that miR-196-5p was significantly upregulated in both CCA cell lines and clinical specimens. In addition, its expression level was proved to be positively correlated with the CCA tumor size, metastasis situation, and TNM stage. Multiple in vitro experiments validated that inhibition of miR-196-5p downregulated the proliferation, migration, and invasion ability of HUCCT1 cells. Mechanically, our results demonstrated that downregulation of HuCCT1 activated Wnt/*β*-catenin signaling pathway in a HAND1-dependent manner. Altogether, our findings suggest the crucial role of miR-196-5p in CCA progression and provide prospective therapeutic targets for CCA treatment.

## 2. Materials and Methods

### 2.1. Data Collection

Two public datasets (GSE140001 and GSE47764) were downloaded from GEO database (http://www.ncbi.nlm.nih.gov/geo). The GSE140001 dataset contained 12 patients with distal CCA and three matched non-neoplastic biliary epithelia samples. The GSE47764 dataset contained 3 human CCA and their corresponding normal bile duct tissues.

### 2.2. Clinical Specimen

This study was approved by the Institutional Review Board and Ethics Committee in Hebei Medical University, and informed consent was obtained from all patients. 56 pairs of CCA tissues and matched adjacent bile duct tissues were collected in CCA resection surgery from March 2019 to December 2020 in The Secondary Hospital of Hebei Medical University. The clinical features of the patients are shown in Table 1.

### 2.3. Cell Culture and Transfection

Human intrahepatic biliary epithelial cell (HIBEC) was obtained from American Type Culture Collection (ATCC) and CCA cell lines including HuCCT1, QBC939, and Huh-28 were preserved in our laboratory. HIBEC and all three CCA cell lines were cultured in RPMI-1640 with 10% FBS (Invitrogen Life Technologies, USA) and 1x penicillin/streptomycin (Gibco, USA) in a humidified air with 5% CO_2_ at 37°C. Among all three CCA cell lines, HuCCT1 exhibited the highest expression level of miR-196-5p; therefore, the HuCCT1 cell line was selected for subsequent functional experiments. MiR-196-5p inhibitor (Inhi-miR-196-5p) as well as inhibitor-nc (Inhi-nc) was acquired from RiboBio (Shanghai, China) and both sequences are protected by patents. The final concentration of Inhi-miR-196-5p or Inhi-nc in the culture supernatant was 100 nM and all functional experiments were performed 48 hours post-transfection. A HAND1 plasmid, which was applied to clone the whole HAND1 sequence, was purchased from GenePharma (Shanghai, China). The sequence details are provided in Table 2. Lipofectamine 3000 (Invitrogen Life Technologies, USA) was used for transfection according to the manufacturer's protocol.

### 2.4. RNA Extraction and RT-qPCR Analysis

Total RNA from all cells was extracted using TRIzol reagent (Invitrogen, CA, USA) according to the manufacturer's protocol. The primer's details for RT-qPCR analysis are provided in Table 2. RT-qPCR analysis was performed using the 7900HT Real-Time PCR Detection System (Thermo Fisher Scientific, USA). The endogenous control U6 (GeneCopoeia, USA) was used for normalization, and the relative expression of miRNAs was calculated using the 2–∆∆Ct method. Each PCR reaction was run in triplicate.

### 2.5. Transwell and Matrigel Invasion Assay

For transwell and matrigel invasion assay, approximately 3 × 10^5^ HuCCT1 and HuCCT1 transfected with miR-196-5p inhibitor or HAND1 plasmids (48 h hours after transfection) were suspended in serum-free medium and seeded into the transwell chambers with 8 *μ*m pore filter insert (Millipore). The medium with 10% FBS was placed into the bottom chamber. To normalize the cell number/viability/proliferation index for the migration/invasion assay, HuCCT1 in all groups was pretreated with 10 *μ*g/ml aphidicolin for 24 h. After 24 h, the cells that had migrated through the membrane were stained with 1% crystal violet for 15 minutes and counted under a light microscope in three random selected fields (200x). Each experiment was repeated three times and for each chamber, three fields were randomly chosen and counted.

### 2.6. Cell Viability and Proliferation Assay

Cell counting kit-8 (CCK-8) (MCE, USA) and Edu staining (RiboBio, China) assays were conducted to assess cell viability and proliferation. Cells in different groups were seeded in 96-well plates with approximately 3000 cells/well in 100 *μ*l medium. 50*μ*l CCK8 was added into wells at 6, 12, 24, 48, and 96 hours, respectively, and incubated for 0.5 h at 37°C according to the manufacturer's protocol. The absorbance was measured at a wavelength of 450 nm. Three independent experiments as well as five replicas were included for analysis at each time point. For EdU proliferation assay, 100 *μ*L 50 *μ*M EdU medium was added into each well and incubated for 2 h in 37°C. The cells were then washed twice with PBS for 10 min, fixed in 4% paraformaldehyde (PFA) for 15 min, neutralized with 2 mg/mL glycine, and washed with PBS before permeabilizing with 0.5% Triton X-100 for 10 min. Finally, the HuCCT1 cells were labelled using 100 *μ*L Apollo-596 staining agent and washed in 0.5% Triton X-100 for three times. Three independent experiments as well as three randomly selected fields were included for analysis.

### 2.7. Colony Formation Assay

Single-cell suspensions in 2 ml of 10% FBS RPMI-1640 containing approximately 400 cells were seeded in six-well plates. After two weeks of culture at 37°C with 5% CO_2_, the cells were fixed in methanol for 30 minutes and stained with 1% crystal violet for 15 minutes at room temperature. Colonies containing 50 cells were counted for subsequent analysis. Each experiment was performed in triplicate.

### 2.8. Western Blot

Cells were lysed on ice for 30 min using RIPA buffer followed by high-speed centrifugation. Afterwards, total cell protein concentration was determined using a BCA Protein Assay Kit (Thermo Fisher Scientific, USA). After blocking, membranes were incubated with antibodies against the desired primary antibodies including Ki67 (ab16667, abcam, USA), E-cadherin (ab40772, abcam, USA), N-cadherin (ab245117, abcam, USA), and GAPDH (10494-1-AP, proteintech, USA). Bands were visualized using enhanced chemiluminescence reagents (NCM, USA) and analyzed using a gel documentation system (Bio-Rad Gel Doc1000 and Multi-Analyst version 1.1). Relative band intensity was determined by densitometry software and normalized with GAPDH protein.

### 2.9. Luciferase Report Assay

Luciferase reporter assay was performed as previously described to explore the potential interaction between miR-196-5p and HAND1 [17]. Briefly, the constructed WT HAND1 and Mut HAND1 vectors were transfected into HuCCT1 cells with miR-196-5p or scramble control in 24-well plates. 48 hours after transfection, dual-luciferase assays were performed using Dual Luciferase reporter assay system according to the manufacturer's instructions.

### 2.10. Immunostaining

To assess the Wnt/*β*-catenin activity, HuCCT1 in different experimental groups was seeded in 24-well plates. After 48 h culture, HuCCT1s were fixed for 30 minutes at room temperature and washed gently for 5 minutes with PBS. Afterwards, cells were permeated and blocked in PBS containing 0.5% Triton X-100, and 5% BSA for one hour before incubating with *β*-catenin antibody (ab32572, abcam, USA) at 4°C overnight. Secondary antibody (SA00013-4, proteintech, USA) was used for signal detection. The immunofluorescence exposure time was normalized (700 ms for both EdU staining and *β*-catenin staining) in all experimental groups, and the images were taken with Olympus FV1000 confocal microscope.

### 2.11. Statistical Analysis

Data are presented as mean ± standard deviation and were analyzed using GraphPad Prism 5.0 software. In all experiments, comparisons between two groups were based on two-sided Student's *t*-test and one-way analysis of variance (ANOVA) was used to test for differences among more groups. *p* value <0.05 was considered statistically significant.

## 3. Results

### 3.1. MiRNA-196-5p Expression Is Upregulated in CCA Specimens and Cells

**Figure 1:**
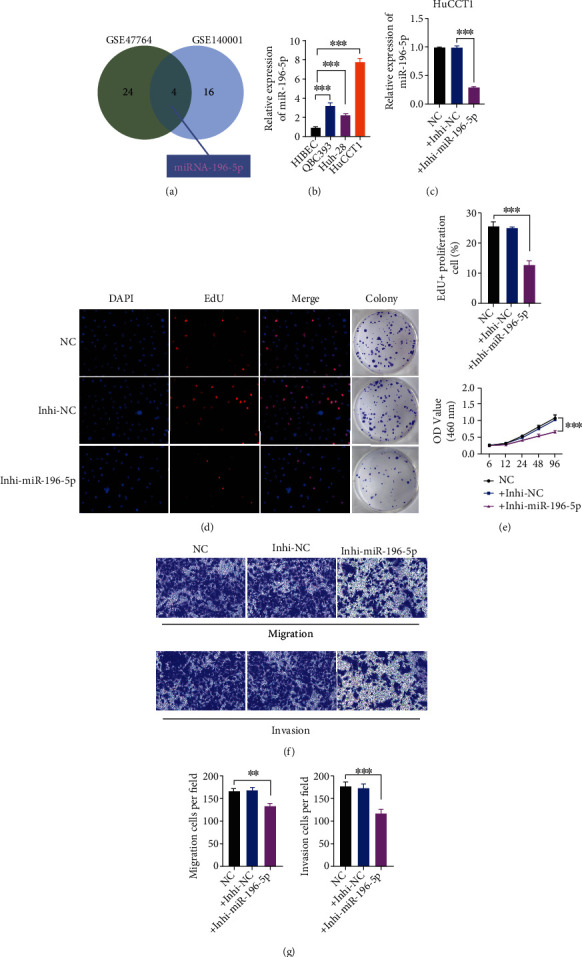
Inhibition of miR-196-5p suppressed cell proliferation and migration in CCA. (a) The intersection of genes upregulated in CCA from the GSE47764 and GSE140001 databases. (b) RT-qPCR results showed the expression of miR-196-5p increased in three different CCA cell lines compared to normal biliary epithelial cell line HIBEC. U6 was used as an internal control for miR-196-5p. Student's *t*-test was used to analyze the statistical significance and all error bars signify standard deviations (*n* = 3). HuCCT1 was chosen for subsequent experiments for miR-196-5p showed the highest increasing rate in it. (c) RT-qPCR verified the transfection efficiency of miR-196-5p inhibitor (Inhi- miR-196-5p). (d) and (e) The effect of Inhi- miR-196-5p on the proliferation capacity of HuCCT1 in vitro was detected using EdU (d [left]), colony formation (d [right]), and CCK-8 assay (e). ∗∗∗ There is a statistical significance between the EdU positive cell percentage in the control group compared to in Inhi-miR-196-5p treated group (*p* < 0.001). Scale bars, 100 *μ*m. (f) and (g) Migration as well as matrigel invasion assay was performed to detect the effect of miR-196-5p depletion on cell migration and invasion in HuCCT1. ∗∗ There is a statistical significance between the migrated cell count in the control group compared to in Inhi-miR-196-5p treated group (*p* < 0.01). ∗∗∗ There is a statistical significance between the invasion cell count in the control group compared to in Inhi-miR-196-5p treated group (*p* < 0.001). Scale bars, 150 *μ*m.

The results of two public datasets (GSE140001 and GSE47764) validated that the expression level of miR-196-5p was elevated in CCA tissues (Figure 1(a)). We further measured the miR-196-5p expression in normal biliary epithelial cell line HIBEC and CCA cell lines including HuCCT1, QBC393, and Huh-28. As shown in Figure 1(b), miR-196-5p expression was notably increased in all CCA cells. Moreover, we analyzed the correlation between miR-196-5p expression and clinicopathological parameters of 56 CCA patients. The miR-196-5p expression was positively correlated with tumor size (*p* = 0.043), metastasis (*p* = 0.010), and TNM stage (*p* = 0.019). However, miR-196 expression did not statistically differ by age and gender (*p* > 0.05, Table 1).

### 3.2. MiR-196-5p Promotes the Proliferation and Migration of CCA

Considering that miR-196-5p expression was positively correlated with tumor size and metastasis, we hypothesized that miR-196-5p may play its oncogenic role by regulating cell proliferation and migration in CCA. To investigate its effects on CCA, HuCCT1 was selected for subsequent in vitro experiments. Inhi-miR-196-5p as well as its scrambled control Inhi-nc was transfected in HuCCT1 and RT-qPCR was utilized to measure transfection efficiency (Figure 1(c)). As shown in Figures 1(d) and 1(e), the results of Edu staining, CCK8, and clonality assay all showed that downregulation of miR-196-5p significantly suppressed the abnormal proliferation capacity in HuCCT1. Additionally, the migration and invasion ability were impaired after miR-196-5p inhibition, as demonstrated by migration and invasion assays (Figures 1(f) and 1(g)). Taken together, our results showed that the relative expression level of miR-196-5p was significantly higher in CCA, and the high level of miR-196-5p might be essential to maintain the tumor proliferation and invasion capacity in CCA.

### 3.3. MiR-196-5p Regulates CCA Function in a HAND1-Dependent Manner

**Figure 2:**
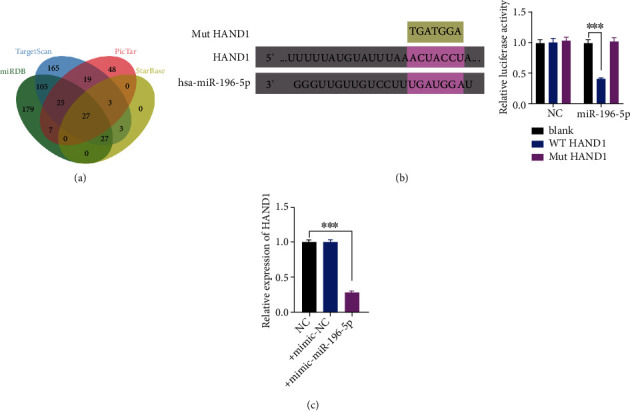
HAND1 is identified as the targeting mRNA of miR-196-5p. (a) Venn diagram depicting the intersection between the miR-196-5p and predictor targeting mRNA. (b) HeLa cells were co-transfected with wild-type plasmid (WT-HAND1) with miR-196-5p or NC and treated mutant plasmid (Mut-HAND1) with miR-196-5p or NC. Luciferase activity was measured 48 hours after transfection. ∗∗∗*p* < 0.001. (c) After treatment with miR-196-5p and NC for 48 hours, the relative expression level of HAND1 was detected by RT-qPCR. ∗∗∗ There is a statistical significance between the expression level of miR-196-5p in the control group compared to mimic-miR-196-5p treated group (*p* < 0.001).

HAND1 was recognized as the potential downstream gene of miR-196-5p using four different predictive tools (Figure 2(a)). To further confirm that miR-196-5p specifically binds to the 3′UTR of HAND1 mRNA, dual-luciferase reporter assay as well as RT-qPCR assay was performed. The results showed that the luciferase activity in the HAND1-WT+miR-196-5p mimic group was robustly downregulated than that in the HAND1-WT+ miR-196-5p group (Figure 2(b)), while there was no significant difference when miR-196-5p mimic was co-transfected with HAND1-MUT. The RT-qPCR results also showed a notable decrease of HAND1 expression when miR-196-5p mimics were transfected into HuCCT1 (Figure 2(c)). Collectively, these findings support the hypothesis that miR-196-5p binds to the 3′-UTR of HAND1 sequence specifically. In addition, the expression level of HAND1 was found to be negatively correlated with TNM stage, tumor size, and metastasis as demonstrated in Table 1.

**Figure 3:**
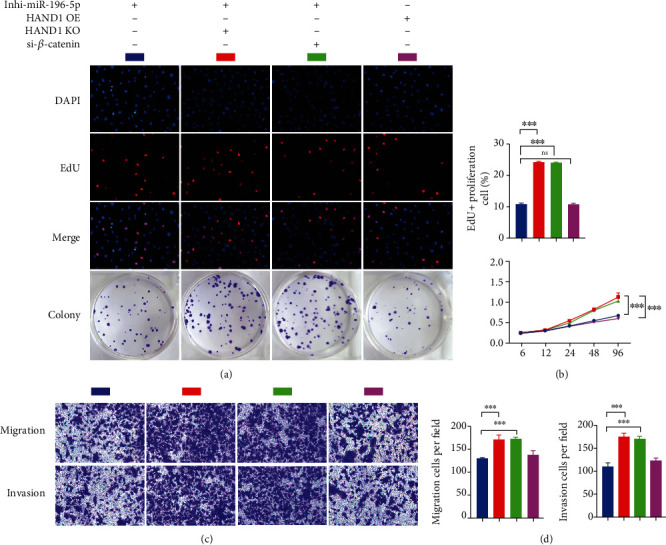
miR-196-5p promotes proliferation and migration of HuCCT1 in a HAND1 and activation manner. (a) and (b) The effect of HAND1 or *β*-catenin depletion on the proliferation capacity of HuCCT1 in vitro was detected using EdU (a [upper]), colony formation (a [lower]), and CCK-8 assay (b). The downregulated cell proliferation of HuCCT1 was reversed by HAND1 knockdown or *β*-catenin silencing. HAND1 OE: HAND1 overexpression; HAND1 KD: HAND1 knockdown. ∗∗∗ There is a statistical significance between the EdU positive cell percentage in blue, compared to red or green group. Scale bars, 100 *μ*m. (c) and (d) Migration and matrigel invasion assays were performed to detect the effect of HAND1 or *β*-catenin depletion on cell migration and invasion in HuCCT1. ∗∗∗ There is a statistical significance between the EdU positive cell percentage in blue, compared to red or green group. Scale bars, 150 *μ*m. Blue panel: Inhi-miR-196-5p, red panel: Inhi-miR-196-5p + HAND1 KD, green panel: Inhi-miR-196-5p+si-*β*-catenin, purple panel: HAND1 OE.

To investigate the role of HAND1 in the regulation of CCA proliferation and migration, HuCCT1 cells were transfected with HAND1 plasmids and functional experiments were performed 48 hours after the transfection efficiency validated by RT-qPCR (Figure 3(a)). We inhibited HAND1 expression in HuCCT1 cells to further confirm the role of HAND1 on cell proliferation and migration. As shown in Figure 3(a), the downregulation of cell proliferation brought by miR-196-5p inhibition was partially reversed after si-HAND1 administration, and similar result was found in both transwell assay and invasion assay (Figures 3(c) and 3(d), Supplement Figure 2). Taken together, these results demonstrated that overexpression of HAND1 is essential to block the effects of miR-196-5p in promoting cell proliferation and migration in CCA.

### 3.4. MiR-196-5p Regulates CCA Progression via Wnt/*β*-Catenin Signaling Pathway

**Figure 4:**
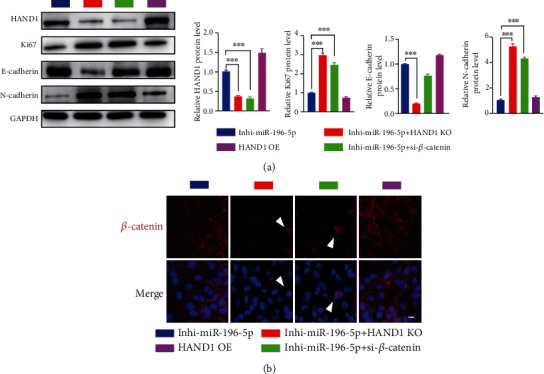
miR-196-5p/HAND1/*β*-catenin axis regulates cell proliferation and EMT process in HuCCT1. (a) Western blot of protein expression of HAND1, proliferation maker Ki67, E-cadherin, and N-cadherin in HuCCT1 with representative images of protein bands for each panel. (b) HuCCT1 was stained with anti-*β*-catenin antibody (red) and DAPI (blue). Pink color indicates the overlap of red and blue colors. White arrows point to the translocation of *β*-catenin expression from membrane to nuclear, which indicates the activation of Wnt/*β*-catenin signaling pathway. Scale bars, 10 *μ*m.

It has been reported that overexpression of HAND1 attenuated tumor metastasis via downregulating *β*-catenin expression [23]; thus, we hypothesized that Wnt/*β*-catenin signaling pathway may be involved in the effects of miR-196-5p/HAND1 axis on CCA behaviors. In vitro experiments including proliferation assay, migration assay, and invasion assay showed that transfection of si-*β*-catenin alleviated the function of miR-196 inhibitor in reducing cell proliferation and migration (Figures 3(a)–3(d)). Also, western blot results showed that the expression level of proliferation marker Ki67 as well as the mesenchymal marker N-cadherin (positively related to cancer metastasis) was significantly suppressed after miR-196-5p inhibition. On the contrary, transfection of both HAND1 and *β*-catenin silencing (si-HAND1 and si-*β*-catenin, respectively) blocked the activation of Wnt/*β*-catenin pathway and had totally adverse biological effects on HuCCT1, as demonstrated by the downregulation of Ki-67- and EMT-related protein expression (Figure 4(a)). Similar results were observed in the immunostaining assays. As shown in Figure 4(b), the merged pictures showed that *β*-catenin was translocated from the nuclei to the membrane after HAND1 or *β*-catenin depletion, also revealing the deactivation of Wnt/*β*-catenin pathway. Altogether, our results demonstrated that miR-196-5p inhibition plays a protective role in CCA in a HAND1/Wnt/*β*-catenin deactivation–dependent manner.

## 4. Discussion

Cholangiocarcinoma (CCA) is a lethal disease including a heterogenous group of biliary malignant tumors originate from the biliary [24, 25]. The incidence of CCA increases rapidly and currently accounts for ~15% of all primary liver cancers worldly [26, 27]. The 5-year survival rate for CCA is <20%, and metastasis and recurrence are the major causes of cancer-related deaths. All current treatments for CCA exhibit significant shortcomings, which emphasizes the need to investigate novel targeting options and provide alternative treatment avenues to improve survival rate of CCA [28–30].

MiR-196 has been reported as a cancer-promoting miRNA that associates with poor prognosis, although not much is known about its role in CCA [16, 17, 31]. Therefore, the identification of miR-196 and the functional significance of its interactions are of interest in CCA progression. In our current study, we investigated the significant role of miR-196-5p in promoting cell proliferation and migration capacity in HuCCT1. MiR-196-5p expression was found to be upregulated in CCA cell lines and was positively correlated with tumor size, metastasis, and TMN staging, indicating a poor prognosis of CCA. In addition, inhibition of miR-196-5p was proven to exert its protective role in a HAND1/Wnt/*β*-catenin–dependent way.

Epithelial-mesenchymal transition (EMT) occurs in multiple physiological and pathological processes especially in tumor progression [32, 33]. In CCA, a series of molecules have been identified as potential targets to mediate EMT process. For instance, Deng et al. once reported that GATA6 can act as an oncogene by promoting metastasis in CCA via activating EMT [34]. In addition, PDGF has been confirmed to upregulate the expression of MMP2/MMP9 and induce EMT by activating the p38/MAPK signaling pathway in CCA [35]. With the rapid development of whole transcriptome sequencing, mounting evidence has identified the crucial role of non-coding RNA in regulating tumor behavior. For instance, Peng et al. recently uncovered the effects and relevant mechanisms of long non-coding RNA CASC2 on cell proliferation and EMT in CCA [34]. We observed a significant positive correlation between miR-196-5p with tumor size, metastasis, and TNM stage and further demonstrated that elevated expression of miR-196-5p can activate EMT process by upregulating N-cadherin and downregulating E-cadherin. By contrast, either miR-196-5p inhibition or overexpress its downstream mRNA HAND1 can totally reverse this alteration. Overall, our work makes a supplement to the regulatory role of miRNA in CCA tumor progression via mediating EMT process.

Moreover, accumulating evidence has revealed the WNT pathway, the well-documented driver of EMT process and metastatic potential, was responsible for invasive behavior and immune escape in multiple cancers [36, 37]. In the light of these findings, significant efforts have been made to the development of effective therapeutics targeting the dysfunctional WNT/*β*-catenin pathway. Our data point out that WNT pathway was activated in CCA cell line HuCCT1 and it is reasonable to speculate that treatments targeting the WNT pathway may yield different outcomes in specific molecular CCA subtypes according to their activation status of WNT pathway. More efforts should be made to detect treatment response including lymph node infiltration status and activation of the immune microenvironment, and to identify candidate drugs to target specific molecular CCA subtypes.

The main limitation of our current work is that we only validated the oncogenic role of miR-196-5p/HAND1 axis in vitro. Our subsequent work would further focus on how would miR-196-5p regulate cell growth as well as invasion in vivo. In conclusion, we confirmed that inhibiting miR-96 expression could restrain the aberrant cell growth and metastasis by restoring the HAND1 expression in HuCCT1. Thus, treatments targeting miR-196 may provide an efficient therapeutic option against CCA.

## Figures and Tables

**Table 1:** Expression levels of miR-196-5p and HAND1 in CCA patients.

Characteristics	Number	miR-196-5p expression			HAND1 expression		
		High group	Low group	*p*	High group	Low group	*p*
Gender							
Female	26	14	12	0.472	11	15	0.410
Male	30	19	11		16	14	
Age (years)							
< 60	24	10	14	0.280	15	9	0.352
≥ 60	32	18	14		16	16	
Tumor size							
< 5 cm	25	7	18	**0.043**	18	7	**0.013**
≥ 5 cm	31	17	14		12	19	
Metastasis							
Present	26	6	20	**0.010**	12	14	**0.016**
Absent	30	17	13		5	25	
TNM stage							
I-II	19	4	15	**0.019**	12	5	**0.003**
III-IV	37	22	15		10	27	

Expression level of miR-196 and HAND1 were correlated with tumor size, metastasis, and TNM stage in 56 CCA patients (chi-square test).

**(a):** 

Gene symbol	
Plasmid	Sequence (5′-3′)
Inhi-NC	CAGUACUUUUGUGUAGUACAA
Inhi-miR-196-5p	CCCAACAACAGGAAACTACCTA
Mimic-NC	GTTVTCCGAACGTGTCACGT
Mimic- miR-196-5p	TAGGTAGTTTCCTGTTGTTGGG

**(b):** 

Primer	Forward	Reverse
HAND1	AAAGGCCCTACTTCCAGAGC	GTCCATCAGGTAGGCGATGT
U6	GCTTCGGCAGCACATATACT	GAGCAGGCTGGAGAA
GAPDH	ACAACTTTGGTATCGTGGAAGG	GCCATCACGCCACAGTTTC

## Data Availability

All data relevant to the study are included in the article.

## Supplementary Materials

Supplementary Materials Supplement Figure 1. The downregulated migration/invasion of Inhi-miR-196-5p transfected HuCCT1 is not a consequence of their decreased proliferation. A 10 *μ*g/ml aphidicolin administration for 24 h did not alter the migration and invasion index of HuCCT1, and the transfection of Inhi-miR-196-5p brought significant downregulation of the migration and invasion capacity compared to its scramble control (Inhi-NC) in the presence of an antimitotic agent, aphidicolin. Scale bars, 150 *μ*m. B Quantification of the migration/invasion index. ∗∗∗*p* < 0.001. Supplement Figure 2. Aphidicolin administration did not alter the effect of HAND1 or *β*-catenin depletion on cell migration and invasion in HuCCT1. Migration and matrigel invasion assays were performed to detect the effect of HAND1 or *β*-catenin depletion on cell migration and invasion in HuCCT1 in the presence of aphidicolin. Scale bars, 150 *μ*m. B Quantification of the migration/invasion index. ∗∗∗*p* < 0.001. Blue panel: Inhi-miR-196-5p, red panel: Inhi-miR-196-5p+HAND1 KD, green panel: Inhi-miR-196-5p+si-*β*-catenin, purple panel: HAND1 OE.
