# Supplementary figures and images for: MiRNA-196-5p Promotes Proliferation and Migration in Cholangiocarcinoma via HAND1/Wnt/β-Catenin Signaling Pathway
Source: J Oncol. 2022 Apr 12;2022:4599676. doi: 10.1155/2022/4599676 (PMC9019430; doi:10.1155/2022/4599676)

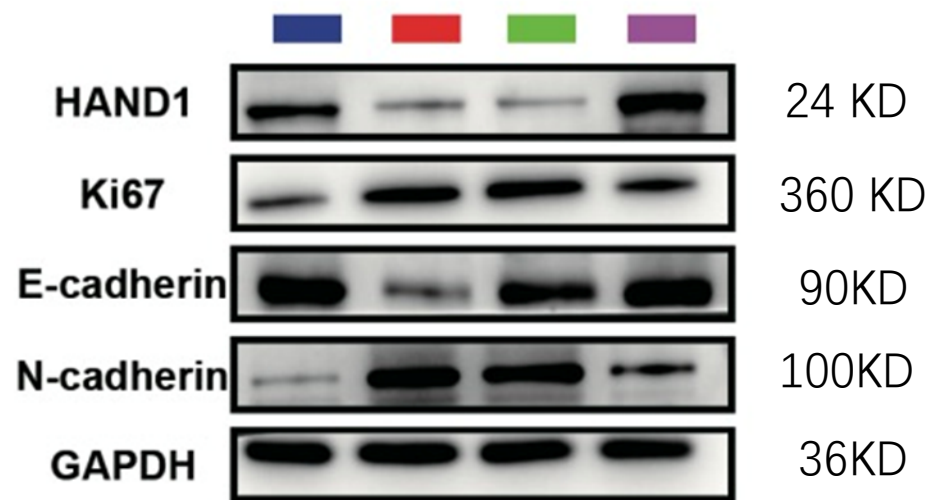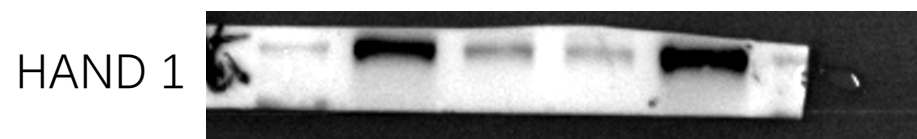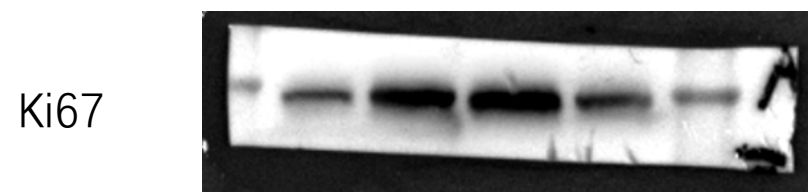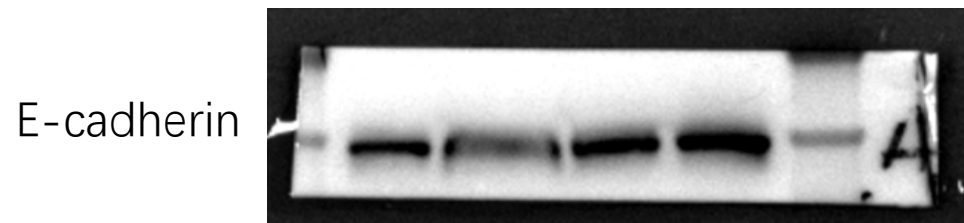

N-cadherin

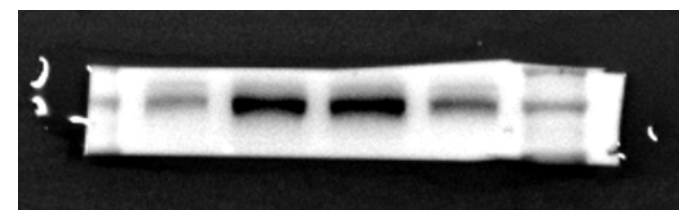

GAPDH

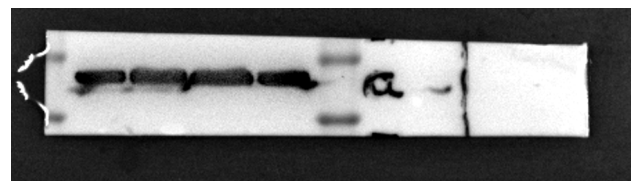

Supplement: Supplementary Materials — Supplement Figure 1. The downregulated migration/invasion of Inhi-miR-196-5p transfected HuCCT1 is not a consequence of their decreased proliferation. A 10 μg/ml aphidicolin administration for 24 h did not alter the migration and invasion index of HuCCT1, and the transfection of Inhi-miR-196-5p brought significant downregulation of the migration and invasion capacity compared to its scramble control (Inhi-NC) in the presence of an antimitotic agent, aphidicolin. Scale bars, 150 μm. B Quantification of the migration/invasion index. ∗∗∗p < 0.001. Supplement Figure 2. Aphidicolin administration did not alter the effect of HAND1 or β-catenin depletion on cell migration and invasion in HuCCT1. Migration and matrigel invasion assays were performed to detect the effect of HAND1 or β-catenin depletion on cell migration and invasion in HuCCT1 in the presence of aphidicolin. Scale bars, 150 μm. B Quantification of the migration/invasion index. ∗∗∗p < 0.001. Blue panel: Inhi-miR-196-5p, red panel: Inhi-miR-196-5p+HAND1 KD, green panel: Inhi-miR-196-5p+si-β-catenin, purple panel: HAND1 OE. [file 4599676.f1.zip › Supplement material.pdf]
